# Supplementary material for: Grazing influences Stipa breviflora seed germination in desert grasslands of the Inner Mongolia Plateau
Source: PeerJ. 2018 Mar 1;6:e4447. doi: 10.7717/peerj.4447 (PMC5835349; doi:10.7717/peerj.4447)
Supplement: Table S1 — Different letters indicate significant differences at P < 0.05. [file peerj-06-4447-s001.docx]

| Seed state | Grazing treatment | Mean ± SE |
| --- | --- | --- |
| Seed with awn intact | Heavy grazing | 52.40 ± 7.96A |
|  | Moderate grazing | 56.00 ± 4.15A |
|  | No grazing | 9.20 ± 2.06B |
| Seed with awn removed | Heavy grazing | 64.40 ± 6.46B |
|  | Moderate grazing | 83.60 ± 3.87A |
|  | No grazing | 44.80 ± 5.64C |
